# Hormonal, chemical and thermal inhibition of spermatogenesis: contribution of French teams to international data with the aim of developing male contraception in France

**DOI:** 10.1186/s12610-016-0047-2

**Published:** 2017-01-13

**Authors:** Jean-Claude Soufir

**Affiliations:** Biologie de la Reproduction, Centre Hospitalier Universitaire Cochin, 123 Bd de Port Royal, 75014 Paris, France

**Keywords:** Male contraception, Spermatogenesis, Epididymis, Testosterone, Progestin, Testicle, Procarbazine, Cyclophosphamide, Irradiation, Gossypol, Heat, Fertility preservation, Contraception masculine, Spermatogenèse, Epididymes, Testostérone, Progestatif, Testicule, Procarbazine, Cyclophosphamide, Irradiation, Gossypol, Chaleur, Protection de la fertilité

## Abstract

Since the 1970s, international research on male contraception has been actively pursued. Hormonal and non-hormonal methods (thermal, chemical) have been tested, leading to clinical trials of interest to thousands of men and couples.

The results showed that it was possible to develop methods of male contraception that inhibited spermatogenesis with good contraceptive efficacy. However, their side effects (mainly loss of libido), poorly accepted modes of administration, and the high frequency of poor responders prevented their widespread use.

Based on earlier initiatives, new avenues were explored and significant progress was achieved, allowing the reasoned use of male contraception. For 40 years, several French teams have played an important role in this research. The aim of this paper is to outline the history and the progress of the experimental and clinical works of these teams who addressed hormonal, chemical and thermal approaches to male contraception. These approaches have led to a better comprehension of spermatogenesis that could be useful in fields other than male contraception: effects of toxic compounds, fertility preservation.

## Abstract in French (Résumé)

Depuis les années 1970, il existe une recherche internationale active sur la contraception masculine. Des méthodes hormonales ou non-hormonales (thermique, chimique) ont été testées, aboutissant à des essais cliniques pouvant intéresser des milliers d’hommes et de couples.

Leurs résultats ont prouvé qu’il était possible de créer des méthodes de contraception masculine inhibant la spermatogenèse avec une bonne efficacité contraceptive. Toutefois, leurs effets secondaires (essentiellement perte de libido), des modalités d’administration mal acceptées, la fréquence élevée de mauvais répondeurs n’autorisaient pas leur diffusion.

A partir de mises au point ébauchées dans le passé, de nouvelles pistes ont été explorées avec des progrès significatifs permettant une pratique raisonnée de la contraception masculine. Depuis 40 ans, plusieurs équipes françaises ont joué un rôle important dans cette recherche. Le présent article a pour objectif de dresser l’historique et les progrès des travaux expérimentaux et cliniques de ces équipes qui se sont intéressées aux approches hormonale, chimique et thermique. Celles-ci ont permis une meilleure compréhension de la spermatogénèse pouvant être utile dans d’autres domaines que celui de la contraception masculine : effets d’agents toxiques, protection de la fertilité.

## Background

Since the 1970s, international research on male contraception has been actively pursued. Several French university teams have taken part in clinical research (development of new hormonal and thermal treatments, participation in two multicenter protocols under the aegis of WHO) and in experimental research (hormonal treatment and its use in protection of the testicle against toxic agents; evaluation of a chemical agent, gossypol, which has been used as a contraceptive in China).

These studies received funding from research organizations: the Institut national de la santé et de la recherche médicale (INSERM), universities, and the World Health Organization (WHO). In civil society they are supported by associations such as the Association pour la Recherche et le Développement de la Contraception Masculine and the Mouvement Français pour la Planification Familiale. Such studies respond to a societal demand which has increased because use of female hormonal contraception has not always been adequately mastered. In this context, two consultations for male contraception were created in France, in Toulouse at the Hôpital Paule-de-Viguier (CHU de Toulouse) and in Paris at AP-HP - CHU Cochin (GHU Paris-Centre). A book aiming to spread knowledge of male contraception was also published [1].

We believed it would be useful to produce a summary report of the results achieved, now that the demand for male contraception is increasing in France (cf. opinion surveys: IFOP 1978, Louis Harris 1991, Institut CSA 2000 [2]) and that some of the results have been implemented in other countries.

## Hormonal contraception

### Clinical research

#### 1976. First trial. Oral progestin and testosterone implants

In the years 1971–1980, encouraged no doubt by the success of female hormonal contraception, several American and Scandinavian teams initiated clinical protocols for male hormonal contraception using steroids (androgens, progestins) [3]. France was not absent from this trend. In 1976, Salat-Baroux and his team [4] carried out the first French trial of male hormonal contraception by combining an oral progestin (R 2323) with testosterone implants. In terms of efficacy, the results were interesting as azoospermia was achieved in 2 to 3 months. The experiment could not be continued because of the development of sexual disturbances (loss of libido, impotence), gynecomastia and weight gain.

Testosterone implants at a dose of 300 mg were insufficient to maintain plasma testosterone at eugonadal levels. Further studies indicated that achievement of eugonadal levels required 400 to 800 mg testosterone implants in combination with progestins administered either orally (desogestrel) [5–7], or as implants (etonorgestrel) [8] or injections (DMPA) [9, 10].

#### Development of a contraceptive treatment using percutaneous testosterone

##### 1950. The French experience of transdermal substance administration

This dates back to the work of Valette and Cavier in 1950 on transdermal absorption of active molecules [11]. Jayle extended this concept to the administration of steroids [12] which was put into practice by the French school of endocrinology: Mauvais-Jarvis, Bercovici, Schaison, and de Lignières [13–16]. Various steroids were tested including testosterone, which had found applications in hematology, hepatology and orthopedics [17].

##### 1978. Development of a contraceptive treatment: percutaneous testosterone-oral progestin

In 1978, faced with a demand for male contraception that arose from the major adverse effects of female contraceptive methods, Soufir’s team responded by proposing a daily treatment consisting of 100 mg testosterone solution (percutaneous testosterone, PT) and oral medroxyprogesterone acetate (MPA) 20 mg, available from pharmacists.

A pilot study in six volunteers demonstrated that, in these conditions, the sperm count reached very low values (−90% at 3 months), that luteinizing hormone (LH) and follicle stimulating hormone (FSH) were equally inhibited and that plasma testosterone remained within the normal range [18, 19]. *For the first time, satisfactory inhibition of spermatogenesis was achieved without elevation of plasma testosterone and without the injection of high doses of steroids.*

In order to better define the effect of the treatment, other subjects were treated with PT alone at the successive doses of 125 mg testosterone for 3 months followed by 250 mg for the next 3 months: although plasma testosterone increased by 30 to 100%, sperm production did not markedly change [20].

Later, the kinetics of inhibition of spermatogenesis, the hormonal profile and the side-effects of the treatment were determined in 35 men and its contraceptive efficacy in 25 couples [21, 22]*.* Spermatogenesis inhibition was accurately measured: sperm concentration was decreased by 47% at 1 month, by 90% at 2 months and by 98 to 100% at 3 months. At 3 months, 80% of men had a sperm concentration of 1 million/mL (M/mL) or less, which is the accepted threshold of contraceptive efficacy [23]; 19% of men already had a sperm concentration <1 M/mL at 1 month and 39% at 2 months. When treatment was discontinued, spermatogenesis rapidly recovered (73 ± 29.5 days) and two couples who wished to have a child had no difficulty in conceiving.

Above all, during treatment, plasma testosterone remained at a physiological level and was maintained throughout the day. Estradiol level was not increased. FSH and LH were rapidly inhibited. Contraceptive efficacy at a sperm count threshold <1 M/mL corroborated the results obtained in the WHO trials (cf. section 4.): 25 couples used this contraceptive method exclusively for 211 months. One pregnancy occurred, due to the fact that the man had discontinued treatment without informing his partner [22].

The combination of MPA-PT was better tolerated than the testosterone enanthate (TE) injections that were used in the WHO trials: it is significant that not a single man stopped treatment for the reasons described in the WHO trials (cf. section 4.) No laboratory parameters were modified, except for a transient moderate increase in hematocrit. However, it was observed that cutaneous application of an alcohol-based testosterone preparation could result in transfer to the partner, and two couples discontinued treatment for this reason. This adverse effect had already been reported elsewhere [24, 25]. It therefore seemed indispensable to clearly define the rules of administration and/or to develop new pharmaceutical forms.

##### 1987–1988. Results of three other university teams. Failures and progress

Two other French teams, led by Guérin and Rollet [25] and by Le Lannou [26], attempted to improve this treatment by changing the type of androgen administered or by using a different progestin. Other authors, Bouchard and Garcia, investigated the use of an LHRH agonist [27].

Guérin and Rollet [25] sought further advances using three treatment modalities:

1. Replacement of PT by percutaneous dihydrotestosterone (DHT) at a dose of 125 mg, in combination with MPA. The results were disappointing: at 3 months, no man had reached the contraceptive threshold (<1 M/mL) and plasma testosterone was markedly low. However, spermatogenesis was satisfactorily inhibited in the same subjects when percutaneous DHT was replaced with PT and at a higher dose (250 mg); of the eight men treated, six became azoospermic and remained so for the entire treatment period. In these subjects, testosterone returned to physiological levels but FSH appeared to be better inhibited than LH.
2. Replacement of PT by oral testosterone undecanoate (TU) at a dose of 160 mg/day: only half of the men became azoospermic and testosterone levels were markedly decreased.
3. Change of progestin: MPA was replaced by norethisterone 5 or 10 mg/day (believed to exert a stronger antigonadotropic effect) while 250 mg PT was continued. The results were excellent: all 13 subjects treated became azoospermic after 2 months treatment. No side effects were observed. With this treatment, LH as well as FSH was perfectly inhibited.

In parallel, Le Lannou’s team [26], disappointed by the variable efficacy of MPA in the first three men treated, used the same progestin as the team of Guérin and Rollet, norethisterone, at a dose of 5 mg/day. Eight of 12 subjects were azoospermic after 6 months treatment.

The third team, Bouchard and Garcia [27], tested the efficacy of long-acting LHRH agonist in ten volunteers; five men received in addition one low monthly dose of TE (125 mg by intramuscular (IM) injection) and the remaining five received a more physiological dose of testosterone (120 mg/day oral TU). The treatment was ineffective as soon as androgen replacement was sufficient: in the first group, 4 of 5 men became azoospermic but spermatogenesis returned as soon as injected testosterone was increased. In the second group, the treatment was ineffective.

#### International impact of percutaneous contraception

Following the French studies, several teams from other countries sought to use the percutaneous approach as a means of contraception.

##### 2001: DHT

Twenty years after the first French publication, Huhtaniemi’s team repeated the same treatment protocol as Guérin and Rollet [25], but the progestin they used was oral levonorgestrel at a dose of 30 microg/day and they doubled the dose of percutaneous DHT (250 mg). However, this did not lead to more convincing results: there was practically no inhibition of spermatogenesis [28].

##### 1999–2002: testosterone patch

During the same period (1999–2002), three teams, the teams of Nieschlag [29], Wu [30], and Wang [31], attempted to replace testosterone gel with a commercial testosterone patch. The patch, renewed daily, was intended to release 5 mg testosterone/24 h in the circulation.

Two studies used a single testosterone patch [29, 30] in combination with oral levonorgestrel (250 then 500 microgr/day) or oral desogestrel (300 microgr/day). The doses of progestin administered were higher than those used in female contraception. In both cases, spermatogenesis was not sufficiently inhibited to ensure effective contraception: in addition, plasma testosterone was unacceptably reduced (−40%).

For this reason, Wang’s team in 2002 [31] increased the dose of testosterone by using two patches, but they prescribed oral levonorgestrel at a lower dose (125 microg/day), similar to that of female contraceptive pills. Inhibition of spermatogenesis was improved, but it was still insufficient: after 3 months treatment, only 15% of subjects had a sperm concentration <1 M/mL. This time, doubling the dose of testosterone maintained plasma testosterone within a physiological range.

##### Rediscovery of the efficacy of testosterone gel. Planned commercialization in the USA

After the failures of DHT gel and patches, 25 years after the first results two teams rediscovered the advantages of administering testosterone as a gel.

Page and colleagues used the same treatment principle (MPA-PT) that had been tested in France, but MPA (depomedroxyprogesterone acetate, DMPA) was given as one injection every 3 months and combined with 100 mg PT/day. They obtained good inhibition of spermatogenesis in 75% of subjects, and sperm concentration was <1 M/ml at 3 months. During treatment, plasma testosterone was increased [32]. Fifty percent of the men who took part in the trial were satisfied with this method and were prepared to use it with their partner [33]. This study also had the merit of showing that use of GnRH antagonists, presented as the male hormonal contraceptive method of the future [34], was not more active than the combination of MPA-PT.

More recently, Wang’s team proposed an “all-in-one” formulation with testosterone and progestin combined in the same gel [35]. The testosterone gel was the same as that used by the French teams. It was combined with nestorone, a new-generation progestin with original properties: it does not bind to the estradiol receptor and its binding affinity with the androgen receptor is 600 times less than that of testosterone, while that of levonorgestrel is 40 to 70% that of testosterone.

Using this combination, 85% of men reached the threshold of contraceptive efficacy at 3 months, with plasma testosterone in the physiological range [35]. These results appeared sufficiently convincing for clinical trials to be launched in the USA in view of commercializing the gel.

##### Mechanisms involved in successful and unsuccessful outcomes

Several explanations have been put forward to explain the unsuccessful outcomes of hormonal treatments: they bear on the hypothalamic-pituitary control of spermatogenesis [36–40], testosterone activation by 5-alpha reductase [41], germ cell apoptosis [42, 43], specific diet [44] and adipose tissue excess [45].

**Fig. 1:**
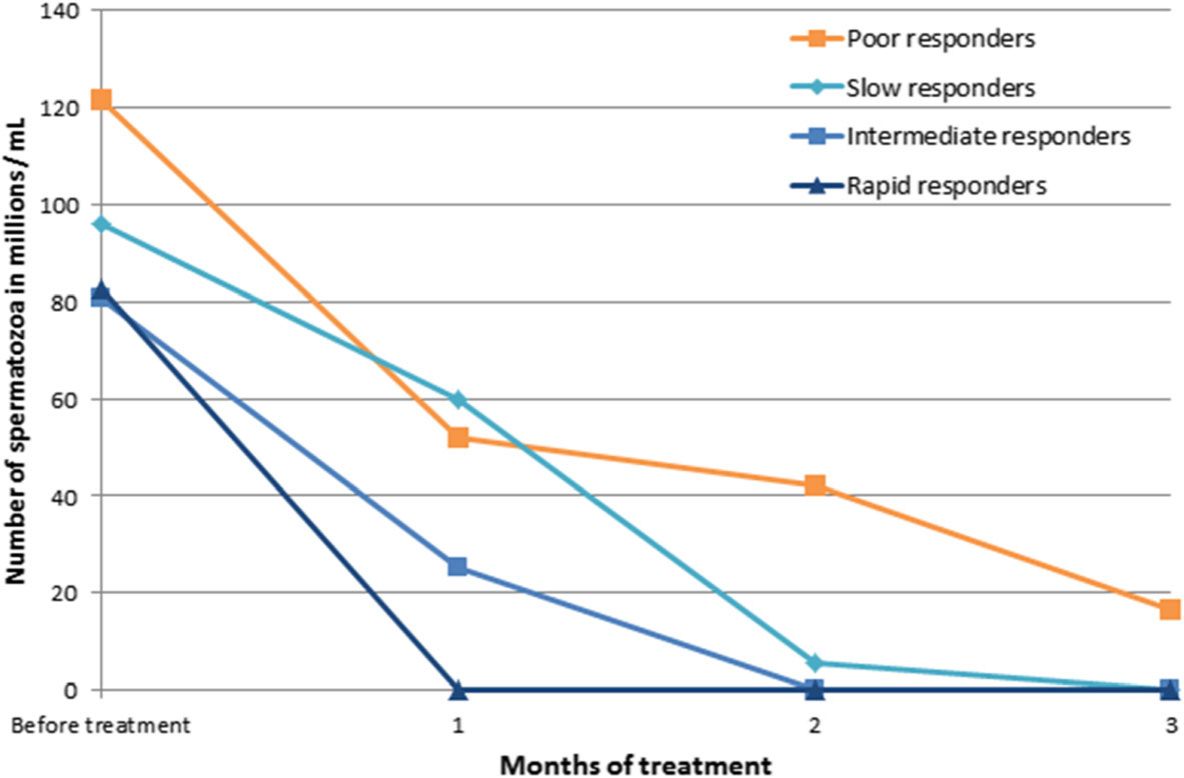
Effect of oral medroxyprogesterone (20 mg/day) and percutaneous testosterone (50–125 mg/day) treatment on sperm count. Number of subjects *n* = 30. Subjects with sperm counts > 1 million/ml at 3 months (*n* = 5) were considered as poor responders, while good responders were subjects with sperm counts < 1 million/ml at 1 month (rapid responders, *n* = 4), 2 months (intermediate responders, *n* = 11) and 3 months (slow responders, *n* = 10)

Studies dealing with the combination of oral MPA and PT are no exception to the rule according to which some men do not sufficiently respond to hormonal treatments. Among 30 men examined 1, 2 and 3 months after the beginning of treatment (using the threshold value of contraceptive efficacy as < 1 M sperm/mL at 3 months), five men were poor responders while the good responders could be divided into 3 types: rapid (*n* = 4), intermediate (*n* = 11) and slow (*n* = 10) according to whether they achieved less than 1 M sperm/mL at month 1, 2 or 3, respectively (Fig. 1) [19, 22].

The azoospermia observed as soon as the first month of treatment strengthens the observations showing that DMPA-PT treatment is able to have a striking effect on spermiation [46]. Besides, the persistence of spermatogenesis in poor responders has been explained by an increased sensitivity of spermatogenesis to high testosterone levels induced by androgen injections [47]. This was not the case for the combination of oral MPA and PT, as no supraphysiological elevation of blood testosterone was induced by this regimen [19, 22]. On the other hand, it may be supposed that the biological availability of oral MPA, which varies greatly from one individual to another [48], could explain the differences observed in response to the oral MPA and PT treatment.

Table 1 presents the results from various teams who used PT (either solution, gel or patches) or percutaneous DHT (gel) in combination with different progestins (MPA, levonorgestrel, desogestrel, norethisterone, nestorone) [19, 22, 25, 28–32, 35]. Two interesting results are apparent from this Table: treatment effectiveness is low when blood testosterone is abnormally low (when testosterone is given as patches) [29–31] or when DHT is the androgen used [25, 28].

**Table 1:** Effects on spermatogenesis inhibition of various progestins combined with either dihydrosterone gel (italic), testosterone patch (underlined), or testosterone in solution or in hydroalcoholic gel formulations (bold)

Authors	Progestin	Route ^a^	Dose	Percutaneous androgen	Dose (mg/day)	Nb ^b^	<1 million/ml at 3 months (%)^c^
DHT gel							
Guérin & Rollet 1988 [25]	MPA	O	20 mg/d ^**d**^	*Andractim* ^*TM*^	*125*	*10*	*0*
Pöllänen et al. 2001 [28]	LN	I	75,150,300 μg/d	*Andractim* ^*TM*^	*250*	*23*	*0*
		O	30 μg/d	*Andractim* ^*TM*^	*250*	*7*	*0*
T patch							
Büchter et al. 1999 [29]	LN	O	250–500 μg/d	Testoderm ^TM^	5	11	0
Hair et al. 2001 [30]	DG	O	75 μg/d	Andropatch ^TM^	5	4	0
			150 μg/d	Andropatch ^TM^	5	6	50
			300 μg/d	Andropatch ^TM^	5	7	70
Gonzalo et al. 2002 [31]	LN	I	160 μg/d	Testoderm ^TM^	10 ^**e**^	20	45
		O	125 μg/d	Testoderm ^TM^	10 ^**e**^	15	30
T solution or gel							
Soufir et al. 1983 [19]	MPA	O	20 mg/d	**PA** ^**f**^	**50–100**	**6**	**80**
Soufir et al. 2011 [22]	MPA	O	20 mg/d	**PA/Testogel** ^**TM**^	**50–125**	**35**	**80**
Guérin & Rollet 1988 [25]	NT	O	5 mg/d	**Testogel** ^**TM**^	**250**	**5**	**100**
			10 mg/d	**Testogel** ^**TM**^	**250**	**5**	**100**
Page et al. 2006 [32]	DMPA	IM	300 mg/3 months	**Testim** ^**TM**^	**100**	**21**	**76**
Ilani et al. 2012 [35]	N	P	8 mg/d	**Androgel** ^**TM**^	**100**	**20**	**85**
			12 mg/d	**Androgel** ^**TM**^	**100**	**19**	**85**

Legend

^a^Route of administration; ^b^number of men; ^c^percentage of men with less than 1 million sperm/mL at 3 months of treatment; ^d^day; ^e^2 patches = 10 mg; ^f^ *PA* percutacrine androgénique^TM^, solution of 100 mg T in 10 mL of 95% alcohol

*DHT* dihydrotestosterone, *MPA* medroxyprogesterone acetate, *LN* levonorgestrel, *T* testosterone, *DG* desogestrel, *NT* norethisterone, *DMPA* depomedroxyprogesterone acetate, *N* nestorone, *O* oral, *I* implant, *IM* intramuscular, *P* percutaneous

It had been shown that DHT (125 mg daily) administered alone reduced blood testosterone from 5.0 to 2.9 ng/mL [49]. The same team had demonstrated that the anti-gonadotropic effect of progestin (even when testosterone-derived) did not involve the androgen receptor but the progesterone receptor, whose expression depends on estradiol originating from testosterone aromatization [50]*.*

These results suggest that the treatment failures responsible for low blood testosterone levels (testosterone patches or DHT gel) or using DHT (which cannot be aromatized) may be explained by a blood testosterone level that is not sufficient to promote an anti-gonadotropic effect of progestin.

#### 1986-90 and 1990-94: two multicenter WHO studies. Contraceptive effectiveness of hormonal treatments

In 1986, the WHO (Task Force on Methods for the Regulation of Male Fertility) undertook two international studies aimed at determining the contraceptive efficacy of an androgen, testosterone enanthate (TE), by IM injection once a week for 18 months.

In seven countries, including France (Soufir, CHU Bicêtre-Université Paris Sud), 271 men with normal semen analysis and in a stable relationship with a partner not suspected of infertility were treated according to this protocol. One hundred fifty-seven men became azoospermic and used this contraceptive method exclusively in their couple. During 1486 months of exposure, only one pregnancy occurred, a Pearl index of 0.8, which is similar to that of female contraceptive pills [51]*.* Treatment with TE did not completely suppress spermatogenesis in 35% of these men: the majority presented with oligozoospermia below 5 M sperm/mL.

This raised a question: what is the lowest sperm concentration required for men to be fertile? A second multicenter study, which began in 1990, established that 3 M sperm/mL appeared to be an acceptable threshold of efficacy [52]. These two protocols also established that less than 2/3 of Europeans have less than 1 M sperm/mL when treated with androgens alone, and that east Asian men are better responders (up to 90%) to these treatments. Efficacy was greater in both Europeans and Asians when androgens were combined with progestins. Spermatogenesis inhibition occurred earlier in Europeans [53, 54].

Various explanations of such ethnic differences have been put forward. Chinese men may have fewer germ cells per Sertoli cell, a higher apoptotic index of germ cells, lower testosterone production with lower plasma testosterone levels, reduced 5 alpha-reductase activity, and LH levels more easily inhibited by testosterone [54].

These results are however not always homogeneous. In a study comparing Europeans from Edinburgh with Chinese men from Shanghai treated with 150 micrograms of desogestrel and a subcutaneous pellet of 400 mg testosterone, treatment seemed more effective in Europeans [6]; a group of Chinese men living in Yunnan were poorer responders to TU probably due to absorption of a local medicinal drink [44]; testosterone or 5 alpha-reductase activity levels did not differ between American Chinese men and American men of Caucasian origin [55].

We may therefore ask whether diet [44] or environment may explain the differences observed between east Asian and European men. The question arises through observations regarding other pharmacological compounds that seemed more active in Chinese than in Caucasian subjects at similar doses [56].

### Experimental studies

#### Contraception using the combination MPA-T in the rat: testicular changes and quality of the descendants after contraception

For better understanding of the effects of the combination MPA-T, Soufir’s team developed an animal model. This treatment administered for 55 days (duration of a spermatogenesis cycle) to adult Sprague-Dawley rats induced a massive decrease in intratesticular testosterone and a particular type of spermatogenesis suppression: the spermatogonia divided normally, but spermatocytes and above all round spermatids decreased by half, while elongated spermatids totally disappeared. This demonstrated that meiosis and above all spermiogenesis are the phases of spermatogenesis that are most sensitive to androgen deficiency.

Seventy days after this treatment, the rats’ fertility returned to normal: litter size was not reduced. There were no fetal resorptions indicative of chromosomal aberrations. The new-borns had no malformations: follow-up of their development in collaboration with Auroux and colleagues showed that behavior did not differ from those of untreated controls [57].

#### Combination of MPA and testosterone: protection of spermatogenesis against cytotoxic agents

Treatment with MPA-T had an unforeseen effect: protection of spermatogenesis against major cytotoxic effects (anticancer drugs, high-dose radiation). The teams of Jégou and Soufir demonstrated this under well-defined conditions (prolonged treatment).

##### Procarbazine [58, 59]

Administered to male rats, procarbazine affects spermatogenesis in both quantity and quality. The genome of the remaining spermatozoa is damaged: the spermatozoa are able to fertilize the oocytes but embryo development (fetal resorptions) as well as postnatal development is affected. This genetic damage is acquired as early as the spermatogonia stage and persists in the descendants.

Prior treatment of rats with MPA-T for 55 days protected spermatogenesis against procarbazine-induced damage. This protective effect concerned both the quantity of spermatozoa produced and their genome.

##### Cyclophosphamide [60]

Male Wistar rats who have been given low-dose cyclophosphamide (10 mg/kg intraperitoneally for 15 days) father litters of normal size. However, their descendants show abnormal behavior at 17 and 21 weeks after birth. This behavior is demonstrated by two tests: the first consists of conditioned reflex learning (shuttle box test), and the second evaluates spontaneous open-field activity. In these conditions, male rats have a decreased success rate and females have reduced spontaneous activity.

Treatment of the male rats with MPA-T (55 days) before administration of cyclophosphamide prevents the appearance of these behavioral disturbances in the offspring.

### Protection against the effects of testicular radiation (3 Gy and 9 Gy). Contradictory results

Testicular irradiation at a dose of 3 Gy causes reduced sperm production and is associated with genome damage of elements of spermatogenesis. This damage is passed on to the next generation (F2 males). In adult rats, short (15 days) as well as long (55 days) pretreatments with MPA-T protect testicular function of irradiated rats [61].

Another study clearly confirmed this protective effect even against stronger doses of radiation (9 Gy). Ten irradiated rats remained permanently sterile. Sterility in rats “protected” by MPA-T treatment was partial: four of ten rats recovered fertility of the same quality as controls [58]. But unfortunately, the protection conferred by treatment of short duration (22 days) did not confirm the protective effect previously described with 15 days treatment, and even appeared to potentiate the effects of radiation [62].

These works benefited from previous results obtained by other teams, in particular Meistrich and his team. The latter identified the site of damage produced by various toxic compounds [63] and demonstrated that GnRH analog did not protect spermatogenesis in mice treated with cyclophosphamide [64]. Meistrich and his team were later able to show that cytotoxic compounds –and more especially irradiation– did not necessarily destroy stem cell spermatogonia, but that the last spermatogonia produced were no longer able to differentiate. Increased FSH levels, and above all excess intratesticular testosterone, explain this phenomenon [65]. Testosterone may act through accumulation of testicular fluid causing edema [66]. However, Leydig cell products that contribute to inhibit spermatogonia differentiation need to be better identified; while its increased expression is correlated with spermatogonial differentiation block, INSL3 does not seem to be involved [67]. This inhibitory effect on spermatogonial differentiation is shared by other androgens (5-alpha DHT, 7-alpha-methylnortestosterone, methyltrienolone) but not by estradiol [68].

Antigonadotropic treatments (GnRH agonists and antagonists, MPA-T) [57–61, 69, 70] induce a protective effect on spermatogenesis in rats This effect does not result from the induction of quiescent spermatogonial stem cells but rather from suppression by testosterone of the block of surviving spermatogonia differentiation. Meistrich and his team also demonstrated that better spermatogenesis recovery was obtained with estradiol than with MPA, while both treatments induced a similar fall in intratesticular testosterone (-98%) [71]. This result could be due to the low androgenic activity of MPA that could explain its relative inefficacy; or rather to the fact that estradiol may increase spermatogonial differentiation through a different mechanism from that which decreases intratesticular testosterone [72].

## Chemical contraception. Gossypol, a male contraceptive agent used in China

### An experimental study

In China, in the province of Jiangxi, physicians had established a causal link between consumption of raw cottonseed oil and the emergence of male infertility. Gossypol, a polyphenolic aldehyde contained in cotton seed, was responsible.

In 1980, 3 years after the end of the Cultural Revolution, the Chinese government decided to use this product as a male contraceptive in 8806 volunteers. In 1990, a Brazilian company announced its intention to commercialize gossypol as a male contraceptive pill.

In 1985, the two teams of Jégou and Soufir undertook experimental research on gossypol using the Sprague-Dawley rat as a model. For the first time, they were able to demonstrate epididymal changes: epididymal secretion was reduced in a dose-dependent manner, epididymal epithelial cells were vacuolized, and spermatozoa were fragmented (head-flagella dissociation, flagellar and hemiaxoneme abnormalities) [73, 74]. These results could open a new approach in the use of gossypol as an epididymal contraceptive. Subsequent studies by the same teams showed that these changes were consecutive to a toxic effect of gossypol on the mitochondria of elongated spermatids which were vacuolized or lysed [75].

One of the surprise findings of these experimental trials was the discovery of a powerful toxic effect: increase of the dose that produced a testicular effect was accompanied by a high fatality rate among the animals. This observation and the notion that gossypol induced severe hypokalemia in healthy volunteers [76] convinced the authors that this molecule could not be used as a safe male contraceptive.

The team of Soufir in collaboration with those of Pointis and Marano completed this research:.they showed that gossypol had a specific effect on Leydig cells: in vitro*,* in the mouse, testosterone production by Leydig cells was increased. This effect was confirmed in vivo: low doses of gossypol stimulated testosterone production, leading to a decrease in LH [77]. They also identified the cellular site of action of gossypol in a flagellated protist (*Dunaliella bioculata*). Gossypol induced swelling of the mitochondria and decreased production of ATP, leading to a fall in motility [78].

## Thermal contraception. Advances by Mieusset’s team

### Clinical research

#### Thermal contraception: history and principle

The discovery of the thermal dependence of spermatogenesis in man dates from 1941 [79]. It was confirmed by experimental studies carried out between 1959 [80] and 1968 [81]. Some authors were already suggesting it might be possible to use an increase in scrotal temperature as a male contraceptive method [80–82]. The contraceptive effect of heat in man was in fact only reported 20 years later by Shafik in 1991 [83].

The increase in temperature was either whole-body heating (steam room at 43 °C, sauna at 77–90 °C) [79, 84, 85], or a high-intensity increase in scrotal temperature (38 to 46 °C) for a short period [80, 82, 86–89], or a low-intensity increase (~1 °C) in scrotal temperature throughout the day [90, 91].

Spermatogenesis was inhibited when thermal elevation was induced by a marked increase in whole-body or scrotal temperature (Table 2), or by a moderate increase in scrotal temperature (Table 3) or in testicular temperature only [83, 92–96] (Table 4), except in a single study using a small temperature increase [91]. These effects on sperm output were associated with decreased sperm motility and altered sperm morphology [80–82, 84, 85, 92, 93, 96, 97].

**Table 2:** Effects of increase in scrotal temperature through high elevation of whole body or scrotal temperature on sperm number in men

Authors	Heating			Nb ^a^	Effect on sperm number			
	Method	Daily duration	Frequency		During heating		After heating	
					Period w ^b^	Mean value	Start w	Max. value
*High elevation of whole body temperature*								
MacLeod & Hotchkiss 1941 [79]	Steam cubicle at 43 °C	45 min OAT ^**c**^ 41 °C	Once	6	w 3–9	50% ^**d**^	w 11	130% ^**e**^ w 15
Procope 1965 [84]	Sauna 77–90 °C	15 min RAT ^**f**^ +1 °C	8 times in 2 weeks	12	w 3–6	60%	w 8	NDA ^**g**^
Brown-Woodman et al. 1984 [85]	Sauna 84 °C	20 min RAT +0.7 °C	Once	5	w 1–4	75%	w 8	NDA
*High-intensity scrotal heating*								
Watanabe 1959 [80]	Scrota in water bath at 44–46 °C	30 min	1 day	5	w 5–10	70%	w 11	158% ^**e**^ w 15
			2 days	4	w 2–10	45%	w 13	NR ^**h**^
			3 days	6	w 3–10	41%	w 11	NR
			6 days	3	w 3–11	47%	w 12	190% ^**e**^ w 14
			12 days	5	w 5–12	42%	w 13	300% ^**e**^ w 16
			Every 2 days for 12 days	4	w 5–11	56%	w 9	240% ^**e**^ w 16
Rock & Robinson 1965 [82]	150 watt lamp at 8 cm; 42 °C	30 min	14 days	4	w 4–8	63%	w 9	177% ^**e**^ w 14
			28 days	3	w 3–9	57%	NDA	NDA
Wang et al. 2007 [86]; Zhu et al. 2010 [87]	Scrota in water bath at 43 °C	30 min	6 days	18	w 3 w 6	(SC) ^**i**^ 56% ^**j**^ 26%	w 9 w 12	(SC) 44% ^**j**^ 109%
Rao et al. 2015 [88]	Lower half body in bathtub at 43 °C	30 min	Every day for 10 days	10	w 4 <5 M/ml 4/10 w 6	(SC) 52% 30%	w8	(SC) w14 106%
			Every 3 days for 30 days	10	w 6 <5 M/ml 4/10 w 8	28% 12%	w10	w16 102%

Legend: ^a^ *Nb* number of men, ^b^ *w* weeks, ^c^ *OAT* oral achieved temperature, ^d^ mean value of total sperm number/initial total sperm number (%), ^e^ maximal value of total sperm number/initial total sperm number (%), ^f^ *RAT* rectal achieved temperature, ^g^ *NDA* no data available, ^h^ *NR* not reported, ^i^ *SC* sperm count, ^j^ maximal value of sperm count/initial sperm count (%)

**Table 3:** Effects of increase in scrotal temperature through scrotal insulation on total sperm count in men

Authors	Heating			Nb ^a^	Effect on sperm number			
	Method	Daily duration	Frequency		During heating		After heating	
					Period w ^b^	Mean value ^c^	Start w	Max. value ^d^
Robinson & Rock 1967 [90]	Insulating (oilcloth) underwear SAT ^**e**^ +0.8 °C	Daytime	Every day for 6 to 10 weeks	10	w 3–9 w 10	<50% [5–20%]	w 1–3 w 10 w 11 w 12–14	<50% 157% 225% 170%
Wang et al. 1997 [91]	Athletic supports with either 1 or 2 layers of P ^**f**^, or 1 P layer plus 1 Al ^**g**^ –impregnated P layer SAT +0.8–1 °C	>20 h	Every day for 24 to 52 weeks	21	No effect whatever the number or type of layers			

Legend: ^a^ number of men; ^b^ weeks; ^c^ mean value of total sperm number/initial total sperm number (%); ^d^ maximal value of total sperm number/initial total sperm number (%); ^e^ *SAT* scrotal achieved temperature; ^f^ polyester; ^g^ aluminum

**Table 4:** Effects of increase in testis temperature (testes in the superficial inguinal pouch) on sperm number in men

Authors	Heating			Nb ^a^	Effect on sperm number			
	Method	Daily duration	Frequency		During heating		After heating	
					Period w ^b^	Mean value	Start w	Max. value
Mieusset et al. 1985 [92]	Testes in the superficial inguinal pouch; special underwear ^**c**^ SAT ^**d**^ +2 °C	15 h	Every day for 6 months	14	w 4 w 12 w 25	54% ^**e**^ 29% 13%	w8 w16 w29 w37	73% ^**f**^ 89% 133% 159%
			Every day for 12 months	8	w 25 to w 52	from 13 to 5%	w10 w16 w37	60% 72% 129%
Mieusset et al. 1987 [93]	Tech 1: same as previous [92] SAT +2 °C	15 h	Every day for 6–24 months	13	9 w 18 w 36 w 45	58% 28% 14% 8%	w9 w18 w36	40% 80% 120%
	Tech 2 = Tech 1 + reinforcement of the hole SAT +2 °C		Every day for 6–24 months	6	w 9 w 18 w 36 w 45	28% 3.5% 4.5% <1%	w9 w18 w36	10% 60% 100%
Shafik 1991 [83]	Testes in the superficial inguinal pouch (stitch suspension technique) SAT +2 °C	Day and night	Every day for 12 months	15 SC ^**g**^ > 40	w 13: 1 < SC ^i^ >10 in 2/15 11 < SC > 20 in 13/15 w 26: SC = 0 in 2/15 0 < SC > 1 in 5/15 2 < SC > 10 in 8/15		NR ^**h**^	NC ^**i**^ w 26: SC > 40 in 15/15
	Ball suspension SAT +2 °C		Every day for 12 months	13 SC > 40	w 13: 1 < SC > 10 in 2/13 11 < SC > 20 in 11/13 w 26: SC = 0 in 2/13 0 < SC > 1 in 5/13 2 < SC > 10 in 6/13		NR	NC w 26: SC > 40 in 13/13
Shafik 1992 [94]	Suspensory sling of polyester fabric ^**j**^ SAT +2 °C	Day and night	Every day for 12 months	14 SC > 40	w 13: SC < 1 in 4/14 2 < SC > 10 in 10/14 w 26: SC = 0 in 14/14		w 5–9	NC w 26: SC > 40 in 14/14
Moeloek 1995 [95]	Suspensory sling as used by Shafik 1992 [94]	Day and night	Every day for 24 weeks	10 SC > 20	w 12: SC < 20 in 4/10 w 24: SC < 20 in 10/10 SC < 10 in 3/10		NR	NR
Ahmad et al. 2012 [96]	Tech 3: Tech 2 [93] + improved material SAT +2 °C	15 h	Every day for 17 weeks	5	w 1 w 3 w 5 w 7 w 10	78% 92% 16% 3.5% 0.5%	w5 w7 w10	2.5% 22% 124%

Legend: ^a^ number of men; ^b^ weeks; ^c^ testes pushed up and maintained in the superficial inguinal pouch by exteriorization of penis and scrotum through a hole in specially designed underwear; ^d^ scrotal achieved temperature; ^e^ mean value of total sperm number/initial total sperm number (%); ^f^ maximal value of total sperm number/initial total sperm number (%); ^g^ sperm count in million/mL; ^h^ *NR* not reported; ^i^ *NC* not calculable; ^j^ with penis uncovered and testes elevated towards the abdomen

The degree of inhibition depended on the level of temperature increase and on its duration. The smaller the range of temperature increase, the longer the daily duration of exposure needed to obtain the same inhibiting effect. Spermatogenesis returned to normal at cessation of temperature elevation.

#### Development of an original technique for elevation of scrotal temperature

Based on these findings, the aim was to develop a practical technique for application of this method that did not interfere with the users’ daily life.

##### Principle

The technique was inspired by the works of Robinson and Rock [90] which had shown that an increase of 1 °C in scrotal temperature could be used as a contraceptive method. However, this slight increase appeared to be inadequate, as the decrease in sperm production did not exceed 80% after 10 weeks. In order to obtain a more marked inhibitory effect, a greater increase of scrotal temperature was required, involving an external source of heat. Moreover, a study in men [98] reported that the temperature of the inguinal canal was about 2 °C higher than that of the scrotum.

In parallel, two reassuring experimental studies on the reversibility of this method were published. In the first study, surgically induced cryptorchidism in the adult dog led to alteration of spermatogenesis that was reversible after return of the testicles in the scrotum [99], while in the second study, local cooling of a naturally cryptorchid testicle in pigs initiated and maintained spermatogenesis leading to complete differentiation in numerous seminiferous tubules [100].

##### Development

Based on these findings and on discussions that took place in 1980 among a group of men who were looking for a male contraceptive method other than withdrawal or condoms, a new technique was developed. The body was used as a source of heat to raise testicular temperature for a sufficiently long period every day. In practice, each testicle was raised from the scrotum to the base of the penis, near the external orifice of the inguinal canal. In this position, elevation of the testicular temperature, estimated at 1.5–2 °C [98], was confirmed by Shafik [83], who detailed in a review the various techniques of induced elevation in testicular temperature that he developed [101].

#### Effects of the technique on sperm production and maturation. Successive adaptations

The testicles were maintained in the required position during waking hours, or 15 h/day, for periods of 6 to 49 months.

##### Model 1

The first procedure was as follows: in close-fitting underwear, a hole was made at the level of the base of the penis. The man passed his penis and then the scrotal skin through the orifice, thus raising the testicles to the desired position. Using this method, in 14 male volunteers followed for 6 to 12 months, both the number and motility of sperm were decreased. Between 6 and 12 months, mean concentration of motile sperm was between 1 and 3 M/mL [92].

##### Model 2

However, this preliminary technique did not ensure that the testicles were maintained constantly in the desired location in all men. A ring of soft rubber was therefore added to the hole in the underwear or was worn alone and held in place by tape. This second technique was evaluated in 6 volunteers (from 6 to 24 months) and it resulted in a more marked effect on spermatogenesis: the total number of motile sperm was reduced by a mean of at least 97% after 2 months, while after the third month, mean concentration of motile sperm was equal to or less than 1 M/mL [93].

##### Model 3

It has been shown that there is a thermal asymmetry between the right and the left scrotum, independently of clothing, position or physical activity [102]. These findings led to the development of a new type of underwear which was more effective than the previous models (less than 1 M motile sperm/mL in 45 to 73 days) [96].

#### Mechanisms of the effects induced by elevation in testis temperature

Molecular mechanisms of testicular heat stress induced by different types of external or internal factors have been reviewed in several recent publications (see for example [103–105]).

Induced elevation of testis temperature for contraceptive purposes is aimed at healthy men in their reproductive life. As shown in Tables 2, 3 and 4, the testis temperature reached ranges from supraphysiological to physiological values. Two of the main advantages of using testis temperature as a male contraceptive are that spermatogenesis can be recovered and fertility is preserved; until now, only physiological increases in testis temperature met such criteria, as spermatogenesis and fertility both recovered after 6 to 24 months of 15 to 24 h/day exposure to +2 °C elevation [83, 106].

In a 15 h/day induced increase (2 °C) in testis temperature, the temperature reached is still within the physiological range. This was not sufficient for most men to achieve azoospermia. Despite the high rate of heat-induced apoptosis [107, 108] some cells – the most heat-vulnerable germ cells, i.e. early primary spermatocytes and early round spermatids in humans [109] – did develop into mature sperm containing damaged DNA, as observed in the inhibitory and recovery phases in 5 healthy volunteers [96]. In this last study of a 15 h/day 2 °C increase in testis temperature for 120 days, on the basis of the literature and of their own results the authors suggest that at the spermatocyte stage some cells underwent apoptosis, some appeared as round cells in the semen, a few continued to develop into sperm and others became arrested in a ‘frozen state’ [96]. As spermatogonia continued dividing and differentiating at the testis temperature reached (only scrotal temperature higher than 42 °C affected mitotic proliferation and the number of spermatogonia [109]) several waves accumulated as late spermatogonia B and spermatocytes in the ‘frozen state’. Finally, when heating was stopped, all arrested germ cells restarted their evolutionary process together, giving a sperm output which began to improve as soon as day 33 after cessation of heating [96]. This could explain why total sperm count values reported after cessation of heating were higher than initial values, whatever the method used to elevate testis temperature, as indicated in the last column of Tables 2, 3 and 4.

#### Contraceptive efficacy

Nine volunteer couples evaluated the contraceptive method developed by Mieusset and colleagues [106]. Three men used the first technique and six the second. The partners of these men discontinued all contraceptive methods after a motile sperm concentration (MSC) of less than 1 M/mL was observed in two successive semen analyses carried out at an interval of 3 weeks. Throughout the duration of the contraceptive period with the first technique, the mean MSC was 1.87 M/mL (range 0 to 7.4) with an MSC below 1 M/mL observed in 41% of sperm analyses performed. Throughout the duration of the contraceptive period with the second technique, azoospermia was observed in 11% of semen analyses and an MSC below 1 M/mL in 86% of analyses.

No pregnancy occurred, except in a single case due to incorrect use of the technique. When temperature increase was discontinued, the MSC returned to the initial values with both techniques [106].

These data obtained between 1985 and 1989 were only published in 1994 [106], after recovery of fertility had been attested. They confirmed the findings of the first study of contraceptive efficacy in men using the method of testicular heating reported by Shafik in 1991 [83]. This researcher used either surgical fixation of the testicles high in the scrotum in 15 men, or the wearing of a cotton sling including two balls that pushed the testes close to the abdomen in 13 other men [83]. In a second study, a polyester sling was used to induce scrotal hyperthermia in 14 men [94]. In both the studies by Shafik, no pregnancies were observed in the 42 couples included in the contraceptive period [83, 94].

In summary, current data from studies evaluating the effect of a moderate increase (1.5 to 2 °C) in testicular temperature induced in men at least during waking hours showed sufficient decrease in the number of sperm and adequate inhibition of their motility to reach the contraceptive threshold. Once this threshold was achieved, contraceptive efficacy was satisfactory in the 50 couples followed over 537 cycles, with occurrence of a single pregnancy due to incorrect use of the technique [83, 94, 106].

#### Other criteria

What of the other criteria that any contraceptive method must meet: acceptability, reversibility and safety?

##### Acceptability

Like all experimental studies, the studies cited are not an evaluation of the acceptability of the thermal method.

##### Reversibility

Inhibition of spermatogenesis is reversible after discontinuation of the method used for periods of 6 to 49 months. Sperm parameters (concentration and motility) returned to normal values in 3 months. In all cases and whatever the technique used, fertility was recovered after discontinuation [83, 94, 106].

##### Safety

Surgical fixation of the testicles [83] carries the risk of potential complications (pain, infection) like any surgical procedure. The wearing of a polyester sling disturbed the men’s sexuality, while blood testosterone levels were unchanged during the period that it was worn [94]. Sexual behavior was assessed before and after 6 and 12 months of wearing the specially designed underwear, and 6 months after it was no longer worn. Behavioral response was rated as potent if the subject’s penis became erect, entered the vagina, and ejaculated. The rate of potent intromission (I) to mounts (M) (I/M ratio) was determined. The changes in sexual behavior were explained as follows: the polyester-containing pants generated electrostatic potentials (EP), as previously reported [110], which may induce electrostatic fields in the intrapenile structures and could explain the diminished sexual activity. Cotton and wool textiles did not generate EP. Thus, polyester underpants could have a detrimental effect on human sexual activity. Six months after their removal, all men recovered their initial sexuality [111].

Use of the underwear developed by Mieusset and colleagues [92, 93, 96, 106] did not cause any of these complications. Recently, this team has shown that sperm nuclear quality was altered during the inhibition phase of spermatogenesis, but that this was reversible 3 months after cessation of hyperthermia [96]. This finding needs to be taken into account when using contraception, during the inhibition phase and for 3 months after discontinuation.

### Experimental research

These clinical trials were completed by experimental studies in rams. A model similar to that used in man was developed in this animal. Testicular temperature (Tp) was increased by 2 °C (normal Tp 32–35 °C, rectal Tp 38–39 °C) by scrotal thermal insulation for 8, 16 or 24 h/day for 30 to 160 days in fertile rams. Semen analyses were carried out weekly and frozen sperm was used for intratubal inseminations that led to pregnancies [112, 113]. Some of the results confirmed the observations made in man: 8 h/day of scrotal insulation did not inhibit sperm production, while inhibition occurred at 16 h/day, and was earlier and more marked at 24 h/day. On the other hand, sperm motility was reduced after 8 h of daily exposure to thermal insulation.

In addition, these experimental studies yielded new data. The fertilization rate of the sperm did not decrease during the first 21 days of exposure, but the rate of embryonic mortality after implantation, indicating abnormal embryo development, increased from the fourth day of thermal insulation. This suggests alteration of the quality of epididymal spermatozoa – probably genomic – right from the beginning of exposure, while other sperm characteristics (number, motility and morphology) remained unchanged [112, 113].

## Conclusion. Toward shared contraception. Commitment is needed from both public bodies and civil society

In France, significant research on male contraception has developed with limited means. It was initiated in parallel in Paris, Lyon, Rennes and Toulouse in response to societal demand from both men and women. This research, whether on hormonal or thermal contraception, has been undertaken in a pragmatic and original manner by physicians anxious to find a response to the need expressed. It led to clinical research in an international context (WHO) and to experimental research funded by INSERM or by universities. Male contraception, which does not bring in large profits compared with the manna that the various hormonal contraceptive methods for women represent for industry, has received little support from the private sector. Elsewhere, countries at the forefront – the USA, China, India – are developing numerous protocols and new molecules are announced too much fanfare in the media.

Commitment by responsible parties in civil society and in public bodies is needed, in moves to activate, pursue and develop the advances made and to evaluate current practices. This would be one of the factors that would contribute to equality between men and women, which is a declared concern of our ministers, and it would encourage research in andrology, singularly deficient in France. Development of this research would help to avoid the undesirable effects of female contraceptive methods that may not be adequately indicated. It would also refine knowledge of male infertility and of its treatments, freeing women from the constraints and complications of medically assisted reproductive techniques for male factors.

This need was very recently expressed during a scientific meeting held on May 4, 2016 in the Paris Academy of Medicine under the initiative of the International Consortium for Male Contraception, of which the author is one of the founding members. On this occasion, the Paris Manifesto on Male Contraception was launched seeking the support of governments and industries [114].

## Abbreviations

DHT: Dihydrotestosterone
DMPA: Depomedroxyprogesterone acetate
DNA: Deoxynucleic acid
FSH: Follicle stimulating hormone
GnRH: Gonadotropin releasing hormone
Gy: Gray
IM: Intramuscular
INSERM: Institut national de la santé et de la recherche médicale
INSL3: Insulin-like factor 3
LH: Luteinizing hormone
LH-RH: Luteinizing hormone-releasing hormone
M/mL: Million sperm per mL semen
microgr: Microgram
MPA: Medroxyprogesterone acetate
MPA-PT: Medroxyprogesterone acetate-percutaneous testosterone
MPA-T: Medroxyprogesterone acetate- testosterone
MSC: Motile sperm count
PT: Percutaneous testosterone
TE: Testosterone enanthate
Tp: Temperature
TU: Testosterone undecanoate
WHO: World Health Organization
